# Proline biosynthesis augments tumor cell growth and aerobic glycolysis: involvement of pyridine nucleotides

**DOI:** 10.1038/srep17206

**Published:** 2015-11-24

**Authors:** Wei Liu, Chad N. Hancock, Joseph W. Fischer, Meredith Harman, James M. Phang

**Affiliations:** 1 Metabolism and Cancer Susceptibility Section, Basic Research Laboratory, Center for Cancer Research, National Cancer Institute at Frederick, National Institutes of Health, Frederick, MD 21702, USA

## Abstract

The metabolism of the nonessential amino acid proline contributes to tumor metabolic reprogramming. Previously we showed that MYC increases proline biosynthesis (PB) from glutamine. Here we show MYC increases the expression of the enzymes in PB at both protein and mRNA levels. Blockade of PB decreases tumor cell growth and energy production. Addition of Δ^1^-pyrroline-5-carboxylate (P5C) or proline reverses the effects of P5C synthase knockdown but not P5C reductases knockdown. Importantly, the reversal effect of proline was blocked by concomitant proline dehydrogenase/oxidase (PRODH/POX) knockdown. These findings suggest that the important regulatory contribution of PB to tumor growth derives from metabolic cycling between proline and P5C rather than product proline or intermediate P5C. We further document the critical role of PB in maintaining pyridine nucleotide levels by connecting the proline cycle to glycolysis and to the oxidative arm of the pentose phosphate pathway. These findings establish a novel function of PB in tumorigenesis, linking the reprogramming of glucose, glutamine and pyridine nucleotides, and may provide a novel target for antitumor therapy.

Tumor metabolic reprogramming driven by nongenetic or gentic factors including oncogenes and tumor suppressors has been recently linked to cancer progression. Besides the Warburg effect, metabolism of nonessential amino acids (NEAA), i.e. glutamine, serine, aspartic acid and proline, has been shown to contribute to tumor metabolic reprogramming^1^,^2^,^3^,^4^,^5^,^6^. Among these, the regulatory functions of proline metabolism proposed 3 decades ago have been recently studied. Of special interest, proline catabolism involving proline dehydrogenase/proline oxidase (PRODH/POX) has been shown to be double-edged sword, which functions either as tumor suppressor to initiate ROS-mediated apoptosis, or as tumor survival factor through ATP production or ROS-induced autophagy depending on the tumor microenvironment^7^,^8^,^9^,^10^,^11^. PRODH/POX itself was regulated by different oncogenic or tumor suppressor signalings, such as p53^12^, PPAR-γ, AMPK^10^, c-MYC (MYC)^9^ etc.

Of all the NEAA, glutamine has received special attention. Besides its contribution to proteins and nucleotides, glutamine through glutamate is a source of α-KG in the TCA cycle, glutathione in redox homeostasis, citrate by reductive carboxylation to form lipids and glucosamines important in the integrity of cell surfaces. A newly appreciated pathway is its conversion to proline through Δ^1^-pyrroline-5-carboxylate/glutamate-γ-semialdehyde (P5C/GSA) catalyzed sequentially by P5C synthase (P5CS) and P5C reductases (PYCRs). We recently showed that MYC reprograms not only glutamine metabolism but also proline metabolism and dramatically increases proline biosynthesis (PB) from glutamine^9^. However, it remains unclear the mechanisms by which the proline biosynthetic pathway fits into the metabolic reprogramming of tumor growth driven by oncogenic signaling. Nevertheless, PYCRs have been intensely studied by several groups of researchers with intriguing findings. These include identification of cutis laxa with PYCR1 deficiency and decreased resistance to oxidant stress^13^, interactions of PYCR with Parkinson protein 7 in Parkinson’s disease^14^ and ORAOV1 gene in esophageal cancer^15^.

In this study, we report that MYC induces PB from glutamine through increasing the expression of the enzymes in PB at both protein and mRNA levels. Furthermore, we document the critical role of PB from glutamine in promoting tumor growth by maintaining pyridine nucleotide levels, connecting the proline cycle to glycolysis and to the oxidative arm of the pentose phosphate pathway.

## Results

### The enzymes in proline biosynthesis were upregulated by oncogenic transcription factor MYC

**Figure 1:**
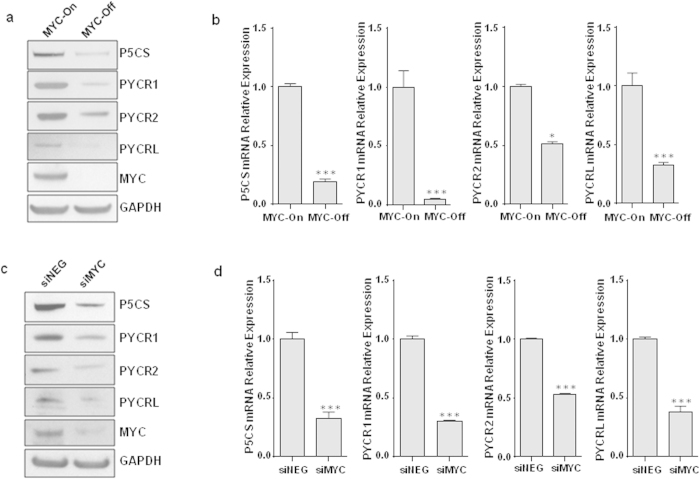
The enzymes in proline biosynthesis were upregulated by MYC. (**a, b**) In P493 human B lymphoma cells, MYC was turned on by removal of tetracycline. (**c, d**) In MCF7 cells, MYC was knocked down by siRNAs (siMYC). Scrambled small RNAs were used as negative controls (siNEG). (**a,c**) The protein expression of P5CS, PYCR1, PYCR2, PYCRL and MYC were detected by western blots. GAPDH was used as a loading control. (**b, d**) The mRNA levels of P5CS, PYCR1, PYCR2, PYCRL were measured by real-time RT-PCR using 18 s rRNA as an internal control. The relative folds were calculated to the group of MYC-On or siNEG. Data shown (mean ± S.E.M., n = 3) represent one of three independent experiments. All *P* values were obtained by two-tailed Student’s *t*-test. ^*^P < 0.05, ^***^P < 0.001 compared with the group of MYC-On or siNEG.

As we previously reported, oncogenic transcription factor MYC markedly increases the biosynthesis of proline from glutamine^9^. MYC increased the expression of glutaminase (GLS), Δ^1^-pyrroline-5-carboxylate (P5C) synthase (P5CS), and P5C reductase-1 (PYCR1) in the proline biosynthetic pathway from glutamine. Since PYCR has three isozymic versions (PYCR1, PYCR2 and PYCRL), in the current study we analyzed both protein and mRNA expressions of P5CS and all 3 PYCR subtypes in response to MYC in P493 human B lymphoma cells bearing a tetracycline-repressible *MYC* construct. As shown in Fig. 1a, when MYC expression was turned on by removal of tetracycline, the protein levels of P5CS and all 3 PYCRs increased markedly. The mRNA expression of these enzymes also significantly increased (Fig. 1b).

Because MYC overexpression plays a critical role in various human cancers, including breast, prostate, and lung cancers, etc., we further tested wether MYC had the same effect on the expression of the above enzymes in MCF7 breast cancer cells by using the short interfering RNA (siRNA) to knock down MYC. As expected, the expressions of P5CS, PYCR1, 2 and L in PB were significantly decreased at both protein and mRNA levels by knockdown of MYC (Fig. 1c,d).

In addition, since PI3K is also a critical mediator of oncogenic signaling in a wide variety of human solid tumors, we investigated the possible effects of PI3K on the expression of the proline biosynthetic enzymes in MCF7. Two widely-used classical PI3K inhibitors, LY294002 and wortmannin, were used to inhibit the phosphorylation of PI3K. As shown in Supplementary Fig. 1a,b, both LY294002 (40 μM) and wortmannin (50 nM) inhibited the protein and mRNA expressions of P5CS, PYCR1, PYCR2, and PYCRL, suggesting that PI3K activitiy regulates the expression of enzymes in PB. However, due to the nonspecific effects of both LY294002 and wortmannin, further investigation is needed.

### Blockade of proline biosynthesis decreased tumor cell growth, but did not affect cell cycle and apoptosis

**Figure 2:**
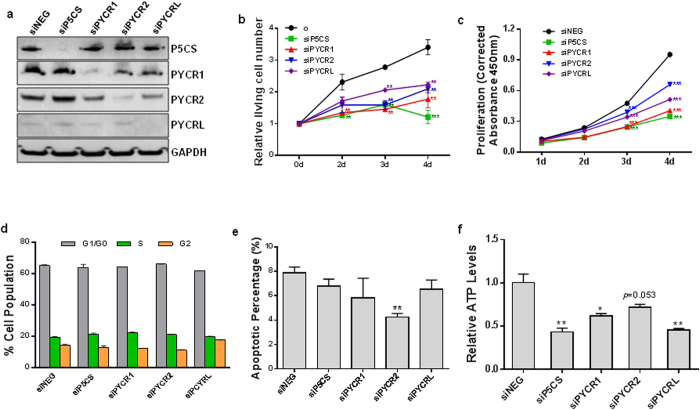
Proline biosynthesis promoted tumor cell growth, but did not affect cell cycle and apoptosis. P5CS, PYCR1, PYCR2, and PYCRL were knocked down by their respective siRNAs (siP5CS, siPYCR1, siPYCR2, siPYCRL) in P493 lymphoma and PC9 lung cancer cells. (**a**) The knockdown of P5CS and three PYCR isozymes was confirmed by western blot with GAPDH as a loading control. Representative western blot images in P493 cells were shown. (**b**) The relative living cell number was determined by trypan blue exclusion assay in P493 cells. (**c**) The cell proliferation assay was performed in PC9 cells. (**d**) On the 4^th^ day after transfection, the cell cycle in P493 cells was determined by propidium iodide (PI) staining using image cytometry. (**e**) Apoptosis in P493 cells was monitored with Annexin V-FITC and PI staining. (**f**) Intracellular ATP production in P493 cells was performed using luciferase-based assay. Data shown (mean ± S.E.M., n = 3) represent one of three independent experiments. *P* values were obtained by two-tailed Student’s *t*-test. ^*^P < 0.05, ^**^P < 0.01, ^***^P < 0.001 compared with the siNEG control group on the same time point.

To investigate whether the proline biosynthetic pathway regulated by oncogenic signalings plays a role in the growth of tumor cells, we knocked down the expression of the enzymes P5CS, PYCR1, 2 and L by their siRNAs in various cancer cell lines, and performed cell proliferation assays or determined the number of living cells. The cell lines we used included P493 lymphoma, PC9 lung, MCF7 and MDA-MB-231 breast, M14 melanoma, and PC3 prostate cancer cell lines. Western blots confirmed the knockdown of P5CS, PYCR1, 2 and L in these cell lines, and Fig. 2a showed a representative image in P493 cells. As shown in Fig. 2b,c, P493 cells and PC9 cells responded in similar fashion to the knockdowns of P5CS, PYCR1, PYCR2 and PYCRL. This was important because P493 cells were in RPMI medium with 0.1 mM proline whereas PC9 cells were in Dulbecco’s MEM without proline. Even with proline at a concentration (0.1 mM) usually used for essential amino acids, the knockdown of proline biosynthesis markedly decreased cell growth. In other cells tested (Supplementary Fig. 2a–d), knockdowns of the aforementioned enzymes inhibited cell growth to various extents. These results suggest that product proline is not the only important endpoint, which was supported by the following data (see below). It was striking that the effect of P5CS knockdown was most significant, and of all the cells tested, P493 and PC9 cells responded most robustly. We thus focused on these two cell lines to explore underlying mechanisms.

An important question is whether the proline biosynthetic pathway decreased tumor cell growth by inducing apoptosis or halting the cell cycle. Unexpectedly, the results showed no obvious effects of the knockdown of these enzymes on either cell cycle or apoptosis in P493 (Fig. 2d,e) or PC9 cells (Supplementary Fig. 2e,f), though there was a small but statistically significant decrease in the apoptotic percentage with PYCR2 knockdown in P493 (7.9% in siNEG vs 4.3% in siPYCR2 in Fig. 2e). However, the production of ATP was significantly decreased by the blockade of PB in both P493 and PC9 cells (Fig. 2f and Supplementary Fig. 2g), suggesting that proline biosynthesis played a role in cellular energy metabolism.

### Proline biosynthesis altered cellular energy metabolism by affecting glycolysis

**Figure 3:**
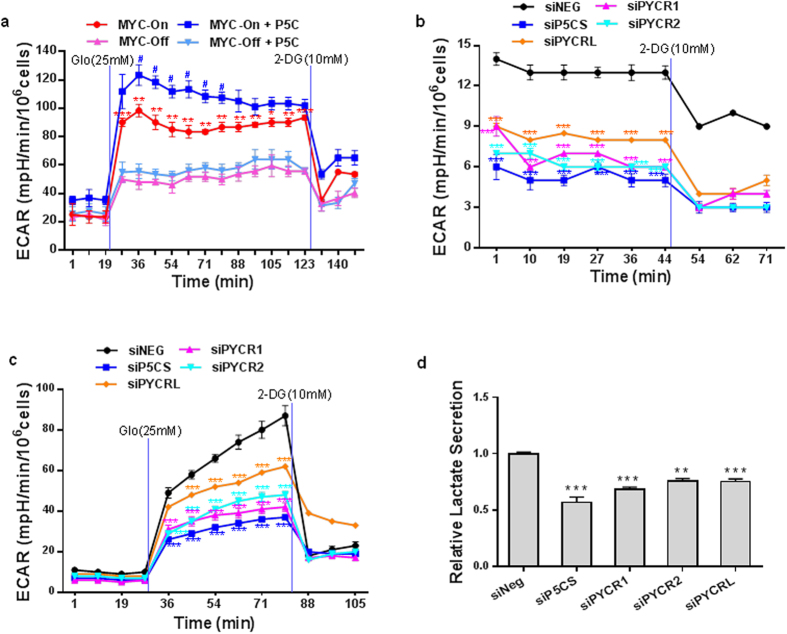
Proline biosynthesis upregulated glycolysis. (**a–c**) P493 lymphoma MYC tet-off cells were cultured under the indicated conditions for 4ds, and extracellular acidification rate (ECAR) were assessed in real time by XF24 flux analyzer. (**a, c**) The cells were deprived of glucose for 2 hr before the assay. (**b**) ECAR was measured under basal conditions after P5CS or PYCR 1, 2 or L was knocked down. (**d**) Analysis of lactate was performed in P493 cell culture supernatants. All measurements were normalized to cell number. The results were from one of three independent experiments. Data shown are mean ± S.E.M. (n = 4). *P* values were obtained by two-tailed Student’s *t*-test. ^*^P < 0.05, ^**^P < 0.01, ^***^P < 0.001 compared with the MYC-Off group, and ^#^*P* < 0.05 compared with MYC-On group at the same time point in 3a. ^**^P < 0.01, ^***^P < 0.001 compared with siNEG control group at the same time point in 3b, c & d.

Considering that MYC upregulated proline biosynthesis as shown above and MYC induces glycolysis, we explored extracellular acidification rate (ECAR), which serves as a marker for quanitating glycolytic flux in cells. Using P493 MYC tet-off cells, we first incubated them for 2 hr without glucose, then measured the reaction of cells to high glucose (25 mM). As shown in Fig. 3a, MYC dramatically increased glycolysis consistent with previous reports^16^,^17^. When P5C was added, glycolytic flux increased significantly only in MYC-On cells. Subsequent addition of 2-Deoxy-D-glucose (2-DG), the glycolysis inhibitor, elicited a rapid decrease of glycolytic fluxes in all cell groups.

We then knocked down the expression of P5CS, PYCR1, 2 or L in P493 cells with MYC-On and measured the basal rate of glycolysis. The blockade of PB markedly inhibited the basal glycolytic rate, especially the knockdown of P5CS (Fig. 3b). The reaction of MYC-On cells to high glucose was also inhibited by knockdown of the enzymes in PB (Fig. 3c). Since ECAR is a nonspecific measure of glycolysis, we used a lactate assay to confirm the results. Knockdown of P5CS or either one of three PYCR subtypes significantly decreased the secretion of lactate (Fig. 3d). In addition, we achieved similar results using PC9 cells by blockading proline synthesis in both basal levels of glycolysis and the response to high glucose (Supplementary Fig. 3a,b).

### Proline biosynthesis forms an interlock between the proline cycle and the recycling of pyridine nucleotides

**Figure 4:**
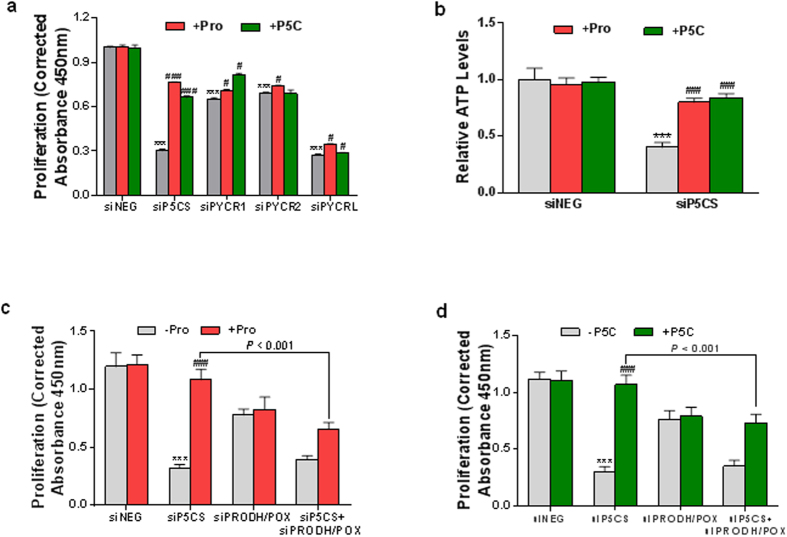
The contribution of proline biosynthesis was linked to proline cycle. PC9 lung cancer cells were cultured for 1–4 ds after the indicated transfection and addition of 0.5 mM proline (Pro) or P5C. (**a,c,d**) The cell proliferation assays were performed at 1–4 ds after transfections. Representative data at 4 ds data were shown. **(b)** ATP production in PC9 cells was performed using luciferase-based assay. Data shown (mean ± S.E.M., n = 3) represent one of three independent experiments. *P* values were obtained by two-tailed Student’s *t*-test. ^***^P < 0.001 compared with siNEG control group. ^#^*P* < 0.05, ^###^*P* < 0.001 compared with “+ Pro” or “+ P5C” group in the same knockdown group.

The growth inhibitory effects of PB knockdown in the face of added proline (Fig. 2b) together with the effects of PB on the glycolytic pathway strongly suggest that proline itself, as the product of PB, is not of primary importance. To elucidate the underlying mechanism(s) of the above effects of PB, we investigated the relationship of proline, the end product of PB, and its intermediate metabolite P5C with cell growth and energy metabolism. The results showed that the addition of both proline (0.1 mM and 0.5 mM) and P5C (0.1 mM and 0.5 mM) in PC9 lung cancer cells markedly reversed the decreased cell growth induced by P5CS knockdown. The concentration of 0.1 mM is close to the serum level of proline in normal humans^18^. As shown in Fig. 4a and Supplementary Fig. 4a,b, 0.1 mM proline and P5C already had significant reversal effects on PB blockade, similar to those of concentration of 0.5 mM. Figure 4a presents the data with 0.5 mM proline and P5C treatment at 4ds, which showed 154% and 119% increase by addition of proline and P5C, respectively, compared with siP5CS group. However, addition of proline or P5C had only minor effects on the decreased cell growth mediated by knockdown of individual PYCR1, 2 or L (0% ~ 25%) though statistically significant. When all three PYCRs were knocked down simultaneously, the effects of proline or P5C addition lost statistical significances (Supplementary Fig. 4c). Similarly, both proline and P5C mitigated the decreased ATP levels due to P5CS knockdown (Fig. 4b). Simultaneous addition of both 0.1 mM proline and 0.1 mM P5C had no additive effect (Supplementary Fig. 4d). We obtained similar results in P493 lymphoma cells (Supplementary Fig. 4e). These results suggested not only the importance of the production of proline from P5C, but also the importance of the production of P5C from proline. The addition of exogenous proline or P5C promotes the interconversion of proline and P5C. It is the metabolic process, i.e. proline cycle, rather than product proline or P5C which underlies the effects of PB on tumor growth.

It has been previously proposed that PRODH/POX and PCYRs mediate the interconversion of proline and P5C to form a “proline cycle” between cytosol and mitochondria^19^. To further confirm the role of the proline cycle on cell growth, we knocked down the expression of PRODH/POX by its siRNAs and examined the mitigating effects of proline or P5C on cell growth inhibition due to P5CS knockdown. As shown in Fig. 4c,d, PRODH/POX knockdown itself decreased cell growth, which is consistent with our previous reports^10^,^11^, suggesting its tumor pro-survival role. Importantly, with PRODH/POX knockdown, the addition of either proline or P5C no longer mitigated the effect of P5CS knockdown. These findings supported the important role of the interconverting of proline and P5C in the proline cycle.

**Figure 5:**
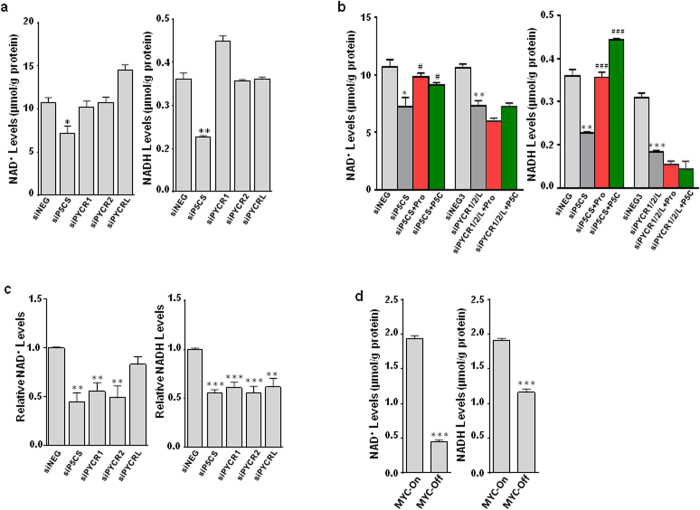
Blockade of proline biosynthesis downregulated NAD^+^ and NADH levels. (**a, b)** PC9 lung cancer cells were transfected with siP5CS, individual siPYCR or all three siPYCRs simultaneously, and cultured under the indicated conditions for 4 ds. The levels of NAD^**+**^ and NADH were measured. (**c, d**) P493 lymphoma MYC tet-off cells were cultured under indicated conditions or transfected with siP5CS or individual siPYCR under MYC-On condition for 4 ds. NAD^+^ and NADH were measured. Data shown (mean ± S.E.M., n = 3) represent one of three independent experiments. *P* values were obtained by two-tailed Student’s *t*-test. ^*^P < 0.05, ^**^P < 0.01, ^***^P < 0.001 compared with the siNEG control or MYC-On group. ^#^*P* < 0.05, ^###^*P* < 0.001 compared with the same knockdown (siP5CS) group in 5b.

As originally proposed, the proline cycle acts as a redox shuttle transferring reducing and oxidizing potentials to maintain redox homeostasis^1^. In this context, the conversion of P5C to proline by PYCRs could result in the recycling of cellular NAD(P)H to NAD(P)^+^, and in addition, the conversion of glutamate to P5C by P5CS also oxidizes NADPH to NADP^+1^. All these reactions could augment glucose metabolism. In Fig. 5a, NAD^+^ and NADH assays showed that both NAD^+^ and NADH were decreased by P5CS knockdown, but not individual PYCR knockdown. This may be due to overlap in function in spite of preferences for specific reduced pyridine nucleotide. As expected, when all three (PYCR1, 2 and L) were knocked down simultaneously, levels of both NAD^+^ and NADH were inhibited significantly (Fig. 5b). Proline and P5C could only recover the effects of siP5CS, but not those due to knockdown of all three PYCRs. Interestingly, we did not find consistent and significant changes in NAD^+^/NADH ratios. Similar results were obtained in P493 cells (Fig. 5c). And as expected, MYC increased both NAD^+^ and NADH levels in these cells (Fig. 5d).

### Proline biosynthesis also linked to pentose phosphate pathway by altering production of NADP^+^ and NADPH in tumor cells

**Figure 6:**
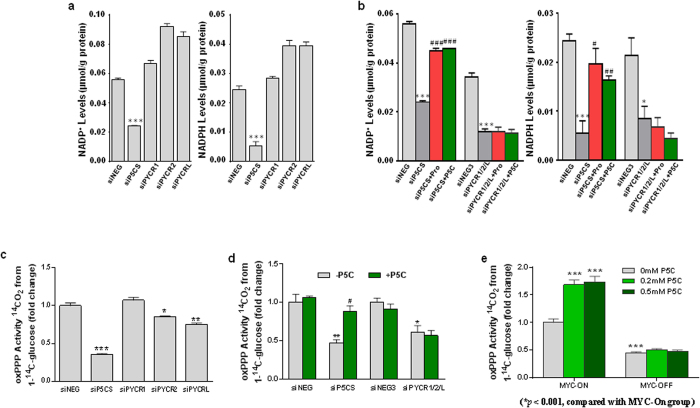
Blockade of proline biosynthesis decreased NADP^+^ and NADPH levels and activities of oxidative arm of pentose phosphate pathway (oxPPP). (**a,b**) PC9 lung cancer cells were transfected with siP5CS, individual siPYCR or all three siPYCR1/2/L simultaneously, and cultured under the indicated conditions for 4 ds. The same amount of scrambled siRNAs (siNEG or siNEG3) was used as negative control. The levels of NADP^+^ and NADPH were measured. (**c,d**) PC9 cells were treated with indicated conditions for 4 ds. Cells were then cultured for 2 hr in the media with 1-^14^C-glucose, and ^14^CO2 were collected for oxPPP activities, which were calculated as 1-^14^C-glucose utilized per hour per mg cell protein. (**e**) P493 lymphoma cells were cultured under indicated conditions for 4 ds, and then oxPPP activities were measured. Data shown (mean ± S.E.M., n = 3) represent one of three independent experiments. *P* values were obtained by two-tailed Student’s *t*-test. ^*^P < 0.05, ^**^P < 0.01, ^***^P < 0.001 compared with the siNEG, siNEG3 or MYC-On without P5C treatment group. ^#^*P* < 0.05, ^###^*P* < 0.001 compared with the same knockdown (siP5CS) group.

The transfer of redox potential in the proline cycle not only is linked to NAD^+^ and NADH, but also NADP^+^ and NADPH^1^. The generation of NADP^+^ has been shown to drive glucose metabolism through the oxidative arm of pentose phosphate pathway (oxPPP). This was shown in fibroblasts and erythrocytes over 3 decades ago. Thus, we wondered whether increased proline biosynthesis in tumor cells also alters the levels of NADP^+^ and NADPH, and the activity of oxPPP. As with NAD^+^ and NADH, the knockdown of P5CS or all three PYCRs markedly decreased the levels of both NADP^+^ and NADPH, but not the ratios of NADP^+^/NADPH (Fig. 6a,b). As expected, only the effects of P5CS knockdown but not those of three PYCRs knockdown were mitigated by the addition of proline or P5C. NADP^+^ is tightly coupled to glucose-6-phosphate dehydrogenase and 6-phosphogluconate dehydrogenase, which together with local channeling in different cellular compartments could be responsible for the failure to detect changes in redox ratios in the whole cell.

We directly tested the activities of oxPPP by measuring ^14^CO_2_ from 1-^14^C-glucose. As shown in Fig. 6c, oxPPP activities were significantly decreased by siP5CS, and moderately by siPYCR2 and siPYCRL alone. Simultaneous knockdown of all three PYCRs were almost comparable to the knockdown of P5CS (Fig. 6d). Consistent with our other results, P5C could only reverse the effects of P5CS knockdown but not those of all three PYCR knockdowns. Using P493 cells, we showed that MYC enhanced the oxPPP activity, while P5C had no effect on this activity when MYC was turned off (Fig. 6e).

### Proline biosynthetic pathway from glutamine but not ornithine was critical for tumor cell growth

**Figure 7:**
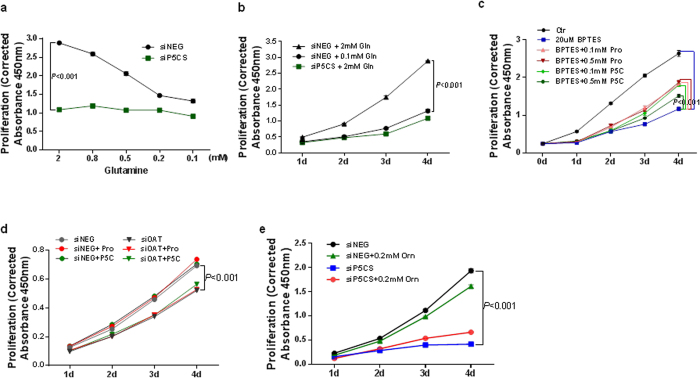
Proline biosynthetic pathway from glutamine but not ornithine was critical for tumor cell growth. PC9 lung cancer cells were cultured under the indicated conditions. (**a–e**) The cell proliferation assays were performed at 1–4 ds after transfections. (**a**) data at 4 ds was shown. Data shown are mean ± S.E.M. (n = 3). The results were repeated three times. *P* values were obtained by two-way ANOVA (time and treatment-dependent changes). Pro, proline; Gln, glutamine; Orn, ornithine; BPTES, a glutaminase inhibitor; OAT, ornithine aminotransferase.

Formation of P5C/GSA from ornithine has been postulated to constitute an alternative pathway of proline biosynthesis and accumulation^20^. Though we have shown the induction of proline synthesis by MYC from glutamine, we couldn’t exclude the possible role of proline biosynthesis from ornithine through ornithine aminotransferase (OAT), and PYCRs. To examine this possibility, we first confirmed the importance of the pathway from glutamine to proline. Fig. 7a showed that glutamine deprivation proportionately decreased cell growth (*P* < 0.001), while with P5CS knockdown, deprivation of glutamine could not further inhibit cell growth, suggesting that the contribution of PB on cell growth was glutamine-dependent. Moreover, when P5CS was knocked down in the face of 2 mM glutamine, cell growth was decreased to levels similar to that with 0.1 mM glutamine (Fig. 7b), suggesting that the effect of glutamine on cell growth was due, in large part, to PB. We further used glutaminase inhibitor BPTES to decrease glutamine degradation and cell growth, and examined whether proline or P5C could mitigate the decrease on cell growth. As previouisly reported^21^, BPTES dramatically inhibited cell proliferation. Interestingly, the addition of either proline or P5C could restore cell growth (Fig. 7c) up to 50% at 4ds, providing further evidence of the importance of PB in tumor growth on glutamine.

Secondly, we observed the effects of OAT knockdown on cell proliferation. As expected, siOAT also decreased cell growth, while neither added proline or P5C could mitigate this effect. This suggests that ornithine produced its effect independent of PB (Fig. 7d). This result was further supported by the inability of added ornithine (0.2 mM) to mitigate the decrease in cell growth produced by P5CS knockdown (Fig. 7e).

## Discussion

Beyond the reprogramming of glycolysis and glutaminolysis, tumor cells upregulate de novo synthesis of several nonessential amino acids. The goal of such reprogramming was supposed to increase the metabolic flux through the pathways to parametabolically promote tumor growth^1^. Previously, Richardson AD *et al.*^22^ had observed de novo proline synthesis as the major metabolic shift in breast metasatic cancer cells. Later, the same group reported increased de novo proline synthesis in melanoma cell lines as compared to melanocytes^23^. Most recently, Vermeersch KA *et al.*^24^ documented that the increased proline level was one of the most important metabolic changes in ovarian cancer cell line OVCAR-3 compared to ovarian cancer stem cells. However, the underlying mechanisms for these aforementioned observations were unclear.

The reprogramming of glutamine metabolism in cancer cells has been intensively investigated^5^,^6^. Glutamine provides energy through the TCA cycle as well as nitrogen and carbon skeletons for nucleotide, amino acid and lipid biosynthesis in growing cancer cells. However, it is less recognized that proline is an important product of glutamine. About 4 decades ago, Stoner G.D and Merchant D.J^25^. showed that during growth of cultured fibroblasts without nonessential amino acids in the medium, the concentration of proline in the medium increased from 0.0 mM at zero time to 0.160 mM by 96 hr. And our previous report^9^ showed that MYC promotes PB from glutamine in cancer cells, and MYC-ON cells have higher intracellular proline levels than MYC-Off cells. Therefore, it is likely that intracellular proline levels would be decreased by knockdown of PB enzymes. In addition, our study also demonstrated the possible effect of PI3K oncogenic signal on PB though additional evidence is needed to confirm the effect. Furthermore, we displayed the critical roles of PB pathway from glutamine in promoting tumor growth. Blocking PB by knockdown of P5CS decreased cell proliferation in the face of 2.0 mM glutamine as much as deprivation of glutamine to 0.1 mM without P5CS knockdown (Fig. 7b). Moreover, the addition of either proline or P5C mitigated the decrease in cell proliferation by glutaminase inhibitor BPTES as much as 50% (Fig. 7c). Taken together, these findings indicate the importance of the PB pathway not only in tumorigenesis but also in the “glutamine addiction” of tumor cells.

Although the augmentation of nonessential amino acids for protein synthesis may contribute to cell growth, our study suggests that PB provides a novel glutamine-derived mechanism(s) for supporting tumor growth. Our interpretation is based on several findings. First, the role of PB on cell growth was independent of added medium proline. For both P493 cells grown in RPMI medium with added proline and PC9 cells grown in Dulbecco’s MEM without proline, the blockade of PB inhibited cell growth. Additionally, knockdown of the enzymes in PB pathway markedly decreased glycolysis measured as extracellular acidification rates on the Seahorse flux analyzer. The addition of medium proline or P5C mitigated the inhibitory effect of P5CS knockdown but not knockdown of PYCRs indicating the importance of the step mediated by the PYCRs. Finally, simultaneous knockdown of PRODH/POX and P5CS abolished the recovery effect of both proline and P5C, confirming the metabolic role of proline cycling.

**Figure 8:**
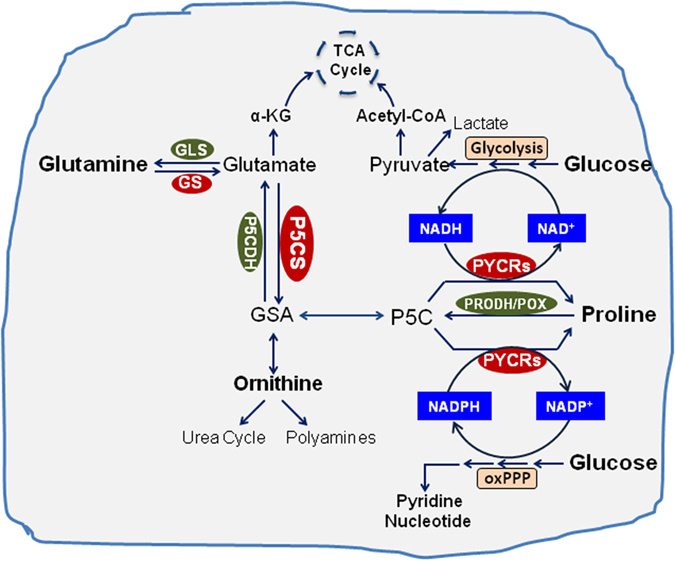
Proposed scheme of interactions of proline biosynthesis with glucose and glutamine metabolism. Proline biosynthesis from glutamine in cancer cells promotes cell growth through interacting with glycolysis and oxidative arm of pentose phosphate pathway. P5C, Δ^1^-pyrroline-5-carboxylate; GSA, glutamic-gamma-semialdehyde; GLS, glutaminase; GS, glutaminesynthase; P5CS, pyrroline-5-carboxylatesynthase; P5CDH, pyrroline-5-carboxylatedehydrogenase; PRODH/POX, proline dehydrogenase/oxidase; PYCR1/2, pyrroline-5-carboxylatereductase1, and 2; PYCRL, pyrroline-5-carboxylatereductase L. oxPPP, oxidative arm of pentose phosphate pathway.

As mentioned above, the proline cycle transfers reducing and oxidizing potentials to maintain redox homeostasis between cytosol and mitochondria through interconversion of proline and P5C catalyzed by PRODH/POX and PYCRs, respectively. In mitochondria, PRODH/POX oxidizes proline to P5C and donates electrons through its flavine adenine dinucleotide into the electron transport chain (ETC) to generate ATP or reactive oxygen species (ROS) for apoptosis or cell growth depending on the metabolic context of tumor environment^10^,^19^. P5C can be converted to proline intra-mitochondrially or in the cytosol by PYCRs using NADPH or NADH as cofactor. Therefore, the interconversion of P5C and proline results in the recycling of cellular NAD(P)H to NAD(P)^+^. During the 1970s and early 1980s, researchers in proline metabolism documented the metabolic interlock between proline cycle and the oxPPP through the cycling of NADPH and NADP^+^ in various cells and reconstituted cell systems^26^,^27^,^28^. They showed that P5C is a potent stimulator of oxPPP in cultured fibroblasts^26^. Together with these early studies, our current findings seen with PB knockdown, i.e. marked decrease in oxPPP activity and decreased levels of both total NAD and NADP, suggest the PYCR-catalyzed conversion of P5C to proline provides a metabolic linkage to oxidize NAD(P)H as well as to generate total NAD and NADP (Fig. 8).

Additionally, PB may also participate in epigenetic regulation in cancer by providing metabolic intermediates or cofactors (NAD^+^) as co-substrate for epigenetic enzymes^1^,^29^,^30^. It has been shown that MYC upregulates NAD^+^-dependent deacetylase SIRT1 (silent information regulator 1) by inducing NAMPT (nicotinamide-phosphoribosyltransferase), the gene encoding the rate-limiting step of NAD^+^ salvage pathway requiring PRPP^31^. This report further increased the possibility that proline biosynthetic pathway is involved in the NAD synthesis and epigenetic modulation. Furthermore, we showed for the first time the connection of PB with glycolysis presumably by affecting the levels of NAD^+^ (Fig. 8). The absence of a shift in NAD^+^/NADH ratios may be due to specific channeling between the NAD^+^ produced by PYCRs and critical steps in glycolysis. In addition, it is notable that the conversion of glutamate to P5C by P5CS also contributes to the oxidation of NAD(P)H to NAD(P)^+^, which may account for the robust effects of siP5CS knockdown on NAD(P)^+^ and NAD(P)H greater than knockdown of all three PYCRs.

Merrill *et al.*^28^ purified human erythrocyte PYCR (subtype unknown) and showed that its *V*_max_ is 10-fold higher but the *K*_m_ for P5C is 7-fold higher with NADH versus NADPH as cofactor. The affinity for NADPH is 15-fold higher (lower *K*_m_) than for NADH. De Ingeniis J *et al.*^32^ addressed the role of three isozymic versions of PYCRs in human melanoma cells by tracking the fate of ^13^C-labeled precursors. They also showed using imaging techniques that PYCR1 and PYCR2 are localized to mitochondria and are primarily involved in conversion of glutamate to proline, while PYCRL is localized in the cytosol and is linked to the conversion of ornithine to proline. In our current study, we did not compare the preference of respective PYCRs for NADH or NADPH as cofactor. However, our results showed that ornithine is not an alternative source for PB in the cancer cells we used. Clearly, the contribution of proline synthesis to tumor growth is based on glutamine as its main source. Wang R *et al.* indicated the importance of ornithine and polyamine biosynthesis from glutamine controlled by MYC in activated T lymphocytes^33^. Here, we did see the inhibitory effect of OAT knockdown on tumor cell growth, but it is not comparable to that with blockade of PB. These results emphasize the specificity of metabolic reprogramming in different tissue and under various metabolic contexts.

It deserves mention that most studies using cultured tumor cells have assigned the nonoxidative arm of the pentose phosphate pathway as the source of ribose and phosphoribosyl pyrophosphate for both salvage and de novo synthesis of purine, pyrimidine and pyridine nucleotides^34^. These findings may be due to specific differences in cells from various tumor sources or due to selection for cells growing at supraphysiologic concentrations of glucose used in tissue culture. Additionally, our studies were performed in cells under stimulated growth conditions. Additional studies are required to dissect out the relative importance of the two arms of the PPP for generating ribose-5-P and PRPP.

In this study, we demonstrated that PB from glutamine is required for tumor cell growth; and we emphasized the participation of the PB enzymes in the cycling of proline and P5C and its parametabolic interlock with glycolysis and oxPPP through recycling of NAD(P)^+^ and NAD(P)H. The latter interlock with oxPPP provides a mechanism for maintaining the content of pyridine nucleotides. Although we didn’t measure the intracellular levels of proline and P5C, which is a limitation in this study, it is unlikely that the parametabolic interlocks can be corroborated by whole cell concentrations of metabolic intermediates. In summary, the study established a critical role for proline metabolism in linking the reprogramming of glutamine and glucose metabolism during tumorigenesis. The enzymes of proline biosynthesis may provide novel targets for cancer therapy.

## Methods

### Cells and cell culture

P493 human B lymphoma cells were cultured in RPMI medium with 10% FBS and 1% pen-strept antibiotics. All other cell lines including PC9 lung, MCF7 and MDA-MB-231 breast, M14 melanoma, and PC3 prostate cancer cell lines, were cultured in Dulbecco’s MEM (DMEM) medium with 10% FBS and 1% Penicillin-Streptomycin antibiotics. The cell culture was maintained in an incubator at 37 °C with 5% CO_2_.

### Small RNAs and cell transfections

The Stealth Select RNAi siRNAs targeting human *P5CS* (designated as siP5CS, target sequences are GGAACACUUCAUGAACUCCUUAGAA, CCCAGGUUCAGAUGAUGCAAAGCUU, and CGGAGAUCAGCAGUCUGUGACAUUU), *PYCR1* (designated as siPYCR1, target sequences are CGGAGGGUCUUCACCCACUCCUACU, GGCGUCUUGGCUGCCCACAAGAUAA, and CAUUGAGAAGAAGCUGUCAGCGUUU)*, PYCR2* (designated as siPYCR2, target sequences are CCACCAUCCACGCCCUGCACUUUCU, GGCAUCCUGUCGGCUCACAAGAUAA, and GCCCUUAAGAAGACCCUCUUAGACA), *PYCRL* (designated as siPYCRL, target sequences are GGUGGCUCCUGUGGUCACCACUGAA, UGCUACAGAGCUGCCUGCUCGUCAU, and GAGGUGCCUGAAGCCUACGUCGACA), *PRODH/POX* (designated as siPRODH/POX, target sequences are UAGAAGGUCAUCUUCAUGAGCUUGU, UAUUCGUGCCACUGCCAUCCCUCUC, and UUCGAUGCAGCGCAAGAAUGUCUCC), *MYC* (designated as siMYC, target sequences are CCAACA

GGAACUAUGACCUCGACUA, GAGAACAGUUGAAACACAAACUUGA, and CAGCGA CUCUGAGGAGGAACAAGAA), *OAT* (designated as siOAT, target sequence is GGGAUUCGACAUCAUUCCCUAUAAA), and Stealth^TM^ RNAi negative controls (designated as siNEG) were purchased from Invitrogen. Transfections of all of the above small RNAs were performed using an Amaxa Nucleotransfection device according to the manufacturer’s instructions. Briefly, 100 nM siRNAs were transfected into 1.0 ~ 2.0 × 10^6^ cancer cells. After 24 h, the cells were treated with conditioned media for certain time and then used for the indicated assays.

### Real-time RT-PCR analysis

Total RNA was harvested using a RNeasy Mini Kit (Qiagen), and the cDNA was synthesized using the SuperScript II Reverse Transcriptase Kit (Invitrogen). Taqman primers for P5CS, PYCR1, 2 and L and internal control 18 s rRNA were purchased from Applied Biosystems.

### Western blot analysis

Cell lysates were prepared in RIPA lysis buffer (Thermo Scientific) and protein was measured by BCA protein assay (Pierce). Equal amounts of cell lysates were electrophoresed on SDS-polyacrylamide gels and transferred by electroblotting onto a nitrocellulose membrane. The primary antibody used were those against MYC, GAPDH (Santa Cruz), PYCR1/2/L, P5CS (Proteintech). Blots were developed using the ECL procedure (Amersham Biosciences). Blots were routinely stripped by the Encore blot stripping kit (Novus Molecular). Anti-rabbit or anti-mouse antibody conjugated to horseradish peroxidase (Santa Cruz) was used as secondary antibody.

### Cell proliferation measurements

The cell proliferation was determined in P493 suspension cells by counting the relative living cell number using trypan blue exclusion assay.

The cell proliferation in other attached cancer cell lines was measured using cell counting kit-8 (Dojindo). The target cancer cells were transfected with siRNAs. At the next day, around 2500 cells were plated into 96-well plates, treated with conditioned media with or without proline, P5C or ornithine. At the test day 10 μl of the water soluble tetrazolium salt (WST-8) from the kit was added to each well containing 100 μl fresh media for 2 hr incubation. The plates were then read at 450 nm in a plate reader.

### Cell cycle analysis

P493 lymphoma and PC9 lung cancer cells were cultured for 4 ds after transfected with siP5CS, or individual siPYCR1, 2, or L. The cells were then harvested from the culture flasks and centrifuged at 1,000 rpm for 5 min. The pellet was resuspended in 200 μl phosphate buffered saline (PBS). The cells were fixed by adding 400 μl of 200 proof ethanol (final percentage at 66%) and incubated for 15 min on ice. The cells were then centrifuged at 1,500 rpm for 5 min and the pellet was resuspended in 200 μl 50 μg/ml propidium iodide (PI) solution (Sigma-Aldrich), 0.1 mg/ml RNase A (Sigma-Aldrich), and 0.05% Triton X-100 (Sigma-Aldrich). The cells were incubated for 40 min at 37 °C and then analyzed for cell cycle by image cytometry using cellometer (Nexcelom).

### Apoptosis assay

The Annexin-V FITC Apoptosis Kit (Invitrogen) was used to moniter the apoptosis of the cells according to the manufacturer’s protocol. Simply, the treated P493 cells or PC9 cells were harvested and washed in cold PBS. Then the cells were re-centrifuged, the supernatant was discarded and cells were resuspended in 1×annexin-binding buffer. 5 μL of FITC annexin V and 1 μL of the 100 μg/mL PI working solution were added into each 100 μL cell suspension. After 15 min incubation, 400 μL of 1× annexin-binding buffer was added to the cells. Then the stained cells were analyzed by flow cytometry immediately.

### Measurement of ATP by luciferase assay

ATP levels were measured by the luciferin/luciferase method using an ATP bioluminescent assay kit (Sigma). After cells were lysed with 1× Passive Lysis Buffer (Promega), 10 μl of lysate was added to the ATP assay reaction mix for a total volume of 110 μl. Luminescence was determined directly using a 20/20 Luminometer (Turner Designs). ATP concentrations were calculated by using an ATP standard curve generated from known concentrations of ATP.

### Glycolysis measurement

P493 lymphoma or PC9 lung cancer cells were cultured in their respective growth medium for 4 ds after transfection with siP5CS or individual siPYCR1, 2, or L. The transfected cells were then seeded in quadruplicate at equal densities (~15,000 cells per well) into XF24 tissue culture plates. At the next day, cell media was changed 2 hr right before the assay into XF assay medium (Seahorse Bioscience Inc.), which is unbuffered DMEM medium adjusted pH to 7.4 according to manufacturer’s protocol. Measurement of glycolytic flux, i.e. extracellular acidification rate (ECAR) was performed during 4-min intervals over the course of 3 hr using the Seahorse XF24 extracellular flux analyzer (Seahorse Bioscience Inc.). During the measurements, the wells were sealed with mechanical plungers containing probes that measure extracellular acidification (pH). ECAR was measured under basal conditions and after injection of glycolytic inhibitor 2-DG (10 mM). ECAR measurements were normalized to cell number in P493 cells, or to protein concentration in PC9 cells which was determined after ECAR measurements.

### Measurement of activity of oxidative arm of pentose phosphate pathway

Cells were cultured for certain time in 25-cm^2^ plastic flask with conditioned growth medium as indicated. On the test day, the medium was removed and replaced with 2 ml of Earle’s balanced salt solution with specifically labeled 1-^14^C-glucose substrate. The flasks were sealed with a serum stopper containing a plastic centerwell (Kontes), and incubated at 37 °C for 2 hours. At the end of incubation, 0.3 ml of 6 N H_2_SO_4_ was injected through the stopper into the medium and the flasks were placed horizontally so that the acidified medium was in contact with the cell monolayer. After 10 minutes of acid treatment, the flasks were placed vertically and 0.3 ml of Hyamine was injected through the stopper into the well. The trapping of carbon dioxide by Hyamine was completed with the flasks in a Dubnoff shaker at room temperature for 45 minutes. The well containing the Hyamine and trapped CO_2_ was transferred into a scintillation vial with 12 ml of Aquasol, 0.2 ml of gracial acetic acid, and the amount of radioactivity was quantitated by liquid scintillation spectrometry.

### Measurement of lactate production

Lactate production was measured by L-Lactate assay kit (Eton Bioscience Inc.) according to the manufaturer’s instructions. Simply, P493 cells were transfected with siP5CS or individual siPYCR1, 2 or L, and then cultured for 4 ds. 50 μl cell culture supernatant or L-lactate standard was added 50 μl L-lactate assay solution in 96-well flat bottom plate. The reaction mixtures were incubated for 30 mins at 37° incubator without CO_2_. 50 μl 0.5 M acetic acid was then added to stop the reaction. The absorbance at 490 nM was measured using a microplate reader. The measurements were normalized to living cell number of each group at the test day.

### Determination of NAD(P)^+^ and NAD(P)H levels

The NAD(P)^+^ and NAD(P)H levels were quantitatively determined according to the protocol described by the manufacturer (BioAssay Systems). NAD^+^/NADH assays and NADP^+^/NADPH are based on a lactate dehydrogenase and glucose dehydrogenase cycling reaction, respectively, in which the formed NAD(P)H reduces a probe into a highly fluorescent product. The fluorescence intensity of the product, measured at λ_ex/em_ = 530/585 nm, is proportional to the NAD(P)^+^ or NAD(P)H concentration in the sample. The NAD(P)^+^ or NAD(P)H content of the cells was harvested separately using a corresponding extraction buffer. The results were normalized to protein contraction.

### P5C synthesis and purification

P5C protected as the dinitrophenyl hydazone was prepared according to the method of Mezl and Knox^35^. Dowex Marathon X2 ion exchange resin in the OH^−^ form was substituted for Dowex 2-OH^−^ resin. ^1^H NMR (400 MHz, DMSO-*d*_6_) δ 11.38 (s, 1H), 8.83 (d, *J* = 2.5 Hz, 1 H), 8.58 (v brs, 3 H), 8.33 (dd, *J* = 9.7, 2.5 Hz, 1 H), 8.04 (t, *J* = 4.3 Hz, 1 H), 7.88 (d, *J* = 9.6 Hz, 1 H), 3.98 (t, *J* = 6.1 Hz, 1 H), 2.71–2.42 (m, 2 H), 2.23–2.01 (m, 2 H,). ^13^C NMR (100 MHz, DMSO) δ 170.71, 152.98, 144.72, 136.62, 129.76, 128.81, 123.04, 116.35, 51.37, 27.91, 26.32.

## Additional Information

**How to cite this article**: Liu, W. *et al.* Proline biosynthesis augments tumor cell growth and aerobic glycolysis: involvement of pyridine nucleotides. *Sci. Rep.* **5**, 17206; doi: 10.1038/srep17206 (2015).

## Supplementary Material

Supplementary Information

## References

[b1] Phang J. M., Liu W. & Hancock C. Bridging epigenetics and metabolism: role of non-essential amino acids. Epigenetics 8, 231–236 (2013). 23422013 10.4161/epi.24042 PMC3669115

[b2] Phang J. M., Liu W., Hancock C. N. & Fischer J. W. Proline metabolism and cancer: emerging links to glutamine and collagen. Curr Opin Clin Nutr Metab Care 18, 71–77 (2015). 25474014 10.1097/MCO.0000000000000121 PMC4255759

[b3] Covini D. *et al.* Expanding targets for a metabolic therapy of cancer: L-asparaginase. Recent Pat Anticancer Drug Discov 7, 4–13 (2012). 21854356 10.2174/157489212798358001

[b4] Possemato R. *et al.* Functional genomics reveal that the serine synthesis pathway is essential in breast cancer. Nature 476, 346–350 (2011). 21760589 10.1038/nature10350 PMC3353325

[b5] Wise D. R. & Thompson C. B. Glutamine addiction: a new therapeutic target in cancer. Trends Biochem Sci 35, 427–433 (2010). 20570523 10.1016/j.tibs.2010.05.003 PMC2917518

[b6] Dang C. V. Glutaminolysis: supplying carbon or nitrogen or both for cancer cells? Cell Cycle 9, 3884–3886 (2010). 20948290 10.4161/cc.9.19.13302

[b7] Liu Y. *et al.* Proline Oxidase Functions as a Mitochondrial Tumor Suppressor in Human Cancers. Cancer Res 69, 6414–6422 (2009). 19654292 10.1158/0008-5472.CAN-09-1223 PMC4287397

[b8] Liu W. *et al.* miR-23b targets proline oxidase, a novel tumor suppressor protein in renal cancer. Oncogene 29, 4914–4924 (2010). 20562915 10.1038/onc.2010.237 PMC4398970

[b9] Liu W. *et al.* Reprogramming of proline and glutamine metabolism contributes to the proliferative and metabolic responses regulated by oncogenic transcription factor c-MYC. Proc Natl Acad Sci USA 109, 8983–8988 (2012). 22615405 10.1073/pnas.1203244109 PMC3384197

[b10] Liu W. *et al.* Proline oxidase promotes tumor cell survival in hypoxic tumor microenvironments. Cancer Res 72, 3677–3686 (2012). 22609800 10.1158/0008-5472.CAN-12-0080 PMC3399032

[b11] Pandhare J., Donald S. P., Cooper S. K. & Phang J. M. Regulation and function of proline oxidase under nutrient stress. J Cell Biochem 107, 759–768 (2009). 19415679 10.1002/jcb.22174 PMC2801574

[b12] Donald S. P. *et al.* Proline oxidase, encoded by p53-induced gene-6, catalyzes the generation of proline-dependent reactive oxygen species. Cancer Res 61, 1810–1815 (2001). 11280728

[b13] Reversade B. *et al.* Mutations in PYCR1 cause cutis laxa with progeroid features. Nat Genet 41, 1016–1021 (2009). 19648921 10.1038/ng.413

[b14] Yasuda T. *et al.* DJ-1 cooperates with PYCR1 in cell protection against oxidative stress. Biochem Biophys Res Commun 436, 289–294 (2013). 23743200 10.1016/j.bbrc.2013.05.095

[b15] Togashi Y. *et al.* Frequent amplification of ORAOV1 gene in esophageal squamous cell cancer promotes an aggressive phenotype via proline metabolism and ROS production. Oncotarget 5, 2962–2973 (2014). 24930674 10.18632/oncotarget.1561 PMC4102783

[b16] Dang C. V. MYC, metabolism, cell growth, and tumorigenesis. Cold Spring Harb Perspect Med 3 (2013). 10.1101/cshperspect.a014217 PMC3721271 23906881

[b17] Mikawa T. *et al.* Dysregulated glycolysis as an oncogenic event. Cell Mol Life Sci 72, 1881–1892 (2015). 25609364 10.1007/s00018-015-1840-3 PMC11113496

[b18] Inoue H., Moritani K., Date Y., Kohashi K. & Tsuruta Y. Determination of free hydroxyproline and proline in human serum by high-performance liquid chromatography using 4-(5,6-dimethoxy-2-phthalimidinyl)phenylsulfonyl chloride as a pre-column fluorescent labelling reagent. Analyst 120, 1141–1145 (1995). 7771678 10.1039/an9952001141

[b19] Liu W. & Phang J. M. Proline dehydrogenase (oxidase) in cancer. Biofactors, 38, 398–406 (2012). 22886911 10.1002/biof.1036 PMC7479541

[b20] Smith R. J. & Phang J. M. The importance of ornithine as a precursor for proline in mammalian cells. J Cell Physiol 98, 475–481 (1979). 438294 10.1002/jcp.1040980306

[b21] Le A. *et al.* Glucose-independent glutamine metabolism via TCA cycling for proliferation and survival in B cells. Cell Metab 15, 110–121 (2012). 22225880 10.1016/j.cmet.2011.12.009 PMC3345194

[b22] Richardson A. D., Yang C., Osterman A. & Smith J. W. Central carbon metabolism in the progression of mammary carcinoma. Breast Cancer Res Treat 110, 297–307 (2008). 17879159 10.1007/s10549-007-9732-3 PMC2440942

[b23] Scott D. A. *et al.* Comparative metabolic flux profiling of melanoma cell lines: beyond the Warburg effect. J Biol Chem 286, 42626–42634 (2011). 21998308 10.1074/jbc.M111.282046 PMC3234981

[b24] Vermeersch K. A., Wang L., Mezencev R., McDonald J. F. & Styczynski M. P. OVCAR-3 spheroid-derived cells display distinct metabolic profiles. PLoS One 10, e0118262 (2015). 25688563 10.1371/journal.pone.0118262 PMC4331360

[b25] Stoner G. D. & Merchant D. J. Amino acid utilization by L-M strain mouse cells in a chemically defined medium. In Vitro 7, 330–343 (1972). 5064338 10.1007/BF02661723

[b26] Phang J. M., Downing S. J., Yeh G. C., Smith R. J. & Williams J. A. Stimulation of the hexose-monophosphate pentose pathway by delta 1-pyrroline-5-carboxylic acid in human fibroblasts. Biochem Biophys Res Commun 87, 363–370 (1979). 36077 10.1016/0006-291x(79)91805-9

[b27] Phang J. M. The regulatory functions of proline and pyrroline-5-carboxylic acid. Curr Top Cell Regul 25, 91–132 (1985). 2410198 10.1016/b978-0-12-152825-6.50008-4

[b28] Merrill M. J., Yeh G. C. & Phang J. M. Purified human erythrocyte pyrroline-5-carboxylate reductase. Preferential oxidation of NADPH. J Biol Chem 264, 9352–9358 (1989). 2722838

[b29] Lu C. & Thompson C. B. Metabolic regulation of epigenetics. Cell Metab 16, 9–17 (2012). 22768835 10.1016/j.cmet.2012.06.001 PMC3392647

[b30] Tasselli L. & Chua K. F. Methylation gets into rhythm with NAD(+)-SIRT1. Nat Struct Mol Biol 22, 275–277 (2015). 25837871 10.1038/nsmb.3004

[b31] Menssen A. *et al.* The c-MYC oncoprotein, the NAMPT enzyme, the SIRT1-inhibitor DBC1, and the SIRT1 deacetylase form a positive feedback loop. Proc Natl Acad Sci USA 109, E187–196 (2012). 22190494 10.1073/pnas.1105304109 PMC3268300

[b32] De Ingeniis J. *et al.* Functional specialization in proline biosynthesis of melanoma. PLoS One 7, e45190 (2012). 23024808 10.1371/journal.pone.0045190 PMC3443215

[b33] Wang R. *et al.* The transcription factor Myc controls metabolic reprogramming upon T lymphocyte activation. Immunity 35, 871–882 (2011). 22195744 10.1016/j.immuni.2011.09.021 PMC3248798

[b34] Patra K. C. & Hay N. The pentose phosphate pathway and cancer. Trends Biochem Sci 39, 347–354 (2014). 25037503 10.1016/j.tibs.2014.06.005 PMC4329227

[b35] Mezl V. A. & Knox W. E. Properties and analysis of a stable derivative of pyrroline-5-carboxylic acid for use in metabolic studies. Anal Biochem 74, 430–440 (1976). 962101 10.1016/0003-2697(76)90223-2

