# Supplementary material for: Proline biosynthesis augments tumor cell growth and aerobic glycolysis: involvement of pyridine nucleotides
Source: Sci Rep. 2015 Nov 24;5:17206. doi: 10.1038/srep17206 (PMC4657043; doi:10.1038/srep17206)
Supplement: Supplementary Information [file srep17206-s1.pdf]

Proline biosynthesis augments tumor cell growth and aerobic glycolysis: involvement of pyridine nucleotides

Wei Liu, Chad N. Hancock, Joseph W. Fischer, Meredith Harman, James M. Phang

Metabolism and Cancer Susceptibility Section, Basic Research Laboratory, Center for Cancer Research, National Cancer Institute at Frederick, National Institutes of Health, Frederick, MD 21702, USA

Supplementary figures

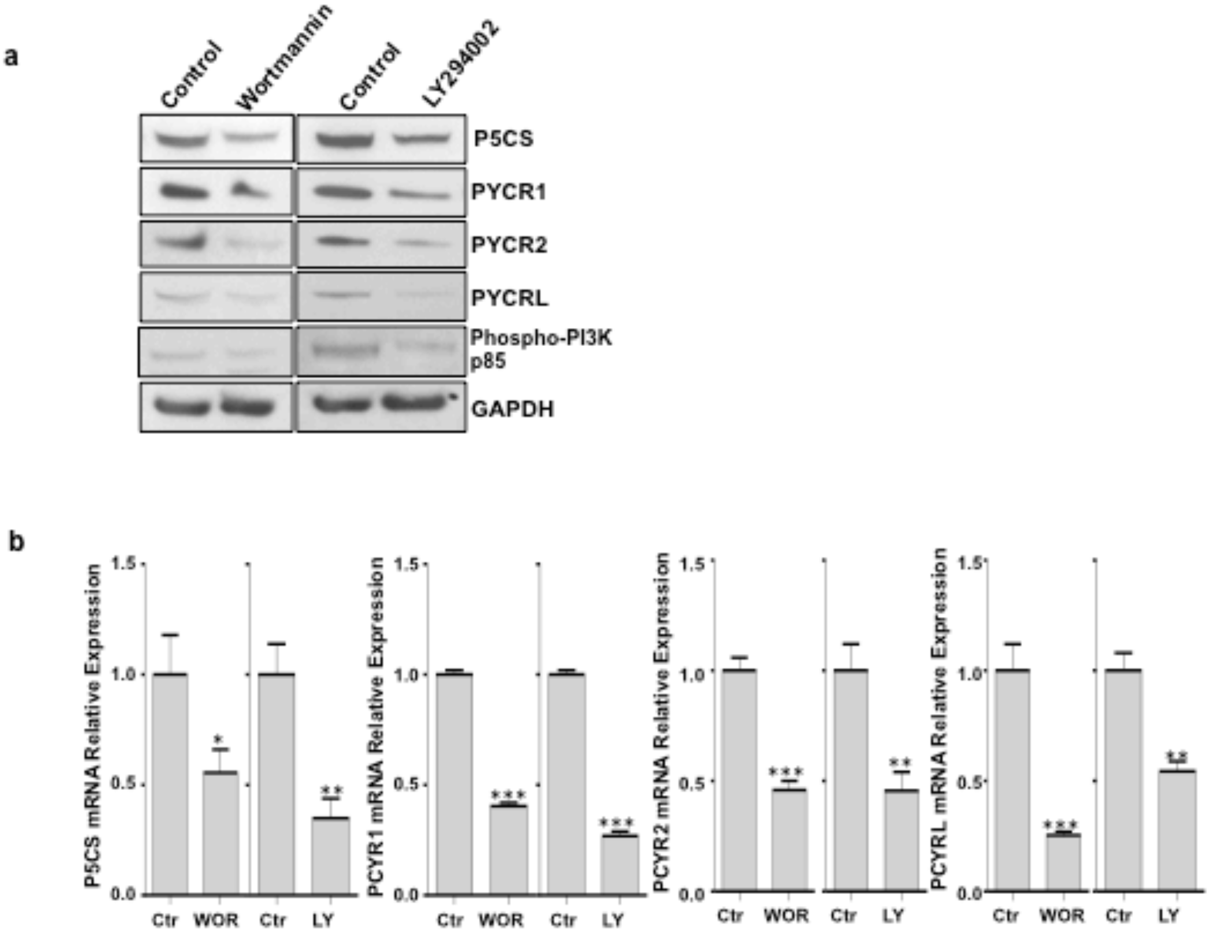

Supplementary Figure 1: The enzymes in proline biosynthesis were upregulated by PI3K oncogenic signaling. (**a**) and (**b**) In MCF7 cells, two classical PI3K inhibitors LY294002 (LY, 40 μM) and wortmannin (WOR, 50nM) were used to inhibit the phosphorylation of PI3K. (**a**) The protein expression of P5CS, PYCR1, PYCR2, PYCRL, and PI3K p85 were detected by western blots. GAPDH was used as a loading control. (**b**) The mRNA levels of P5CS and all three PYCRs were measured as described above. The relative folds were calculated to the control (Ctr) group. All western blot data was repeated at least twice. The mRNA results shown are mean ± S.E.M., n=3. *P* values were obtained by two-tailed Student's *t*-test. $^{*}P<0.05$, $^{**}P<0.01$, $^{***}P<0.001$ compared with the control group.

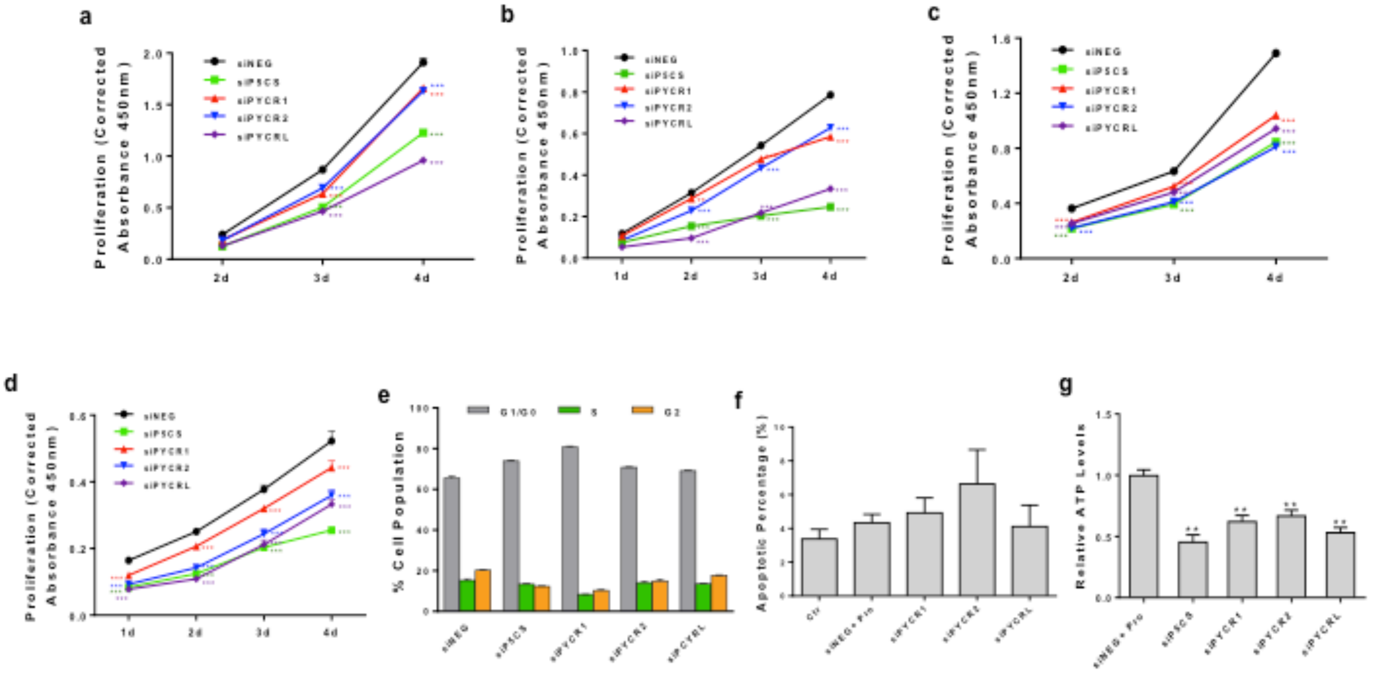

Supplementary Figure 2: Proline biosynthesis promoted tumor cell growth, but not affected cell cycle and apoptosis. The cell proliferation assays were performed in breast cancer cell lines MCF7 (**a**) and MDA-MB-231 (**b**), M14 melanoma cell line (**c**), and PC3 prostate cancer cell line (**d**), after P5CS, PYCR1, PYCR2, and PYCRL were knocked down by their respective siRNAs in those cells. (**e**) On the 4th day after transfection, the cell cycle in PC9 lung cancer cells was determined by propidium iodide (PI) staining using image cytometry. (**f**) Apoptosis in PC9 cells was monitored by Annexin V-FITC and PI staining. (**g**) Intercellular ATP production in PC9 cells was performed using luciferase-based assay. Data shown (mean ±S.E.M., n=3) represent one of three independent experiments. *P* values were obtained by two-tailed Student's *t*-test. $^{**}P<0.01$, $^{***}P<0.001$ compared with the siNEG control group.

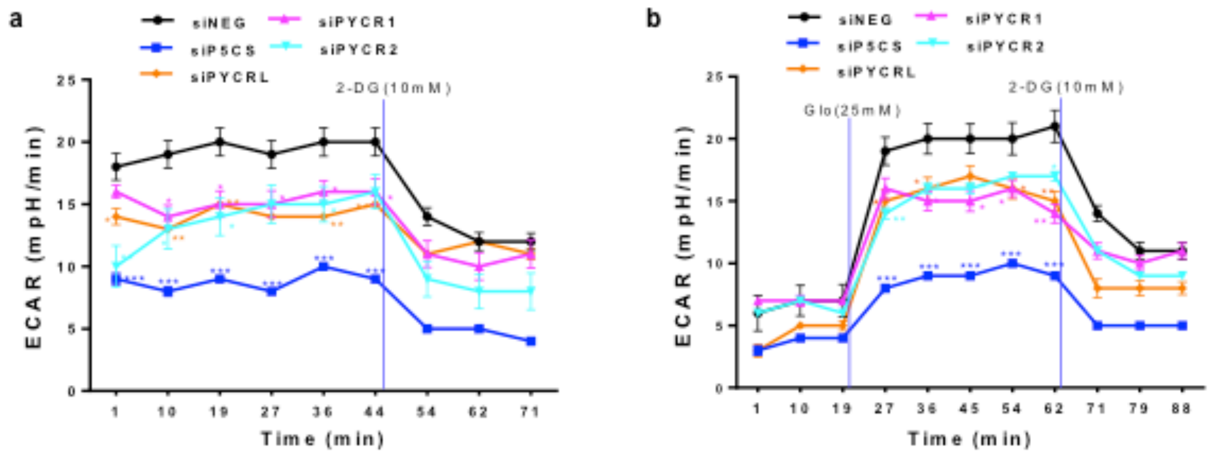

Supplementary Figure 3: Proline biosynthesis upregulated glycolysis in PC9 lung cancer cells. PC9 cells were cultured for 4ds after transfected with siP5CS or isozymatic PYCR 1, 2 or L. (**a**) Basal level of extracellular acidification rate (ECAR) were assessed in real time by XF24 flux analyzer. (**b**) The cells were deprived of glucose for 2hr right before the assay, and the glycolytic reactions of each group to high glucose (25mM) were monitored. The measurements were normalized to protein concentrations. Data shown (mean ±S.E.M., n=4) represent one of three independent experiments. *P* values were obtained by two-tailed Student's *t*-test. $^{*}P<0.05$, $^{**}P<0.01$, $^{***}P<0.001$ compared with siNEG control group at the same time point.

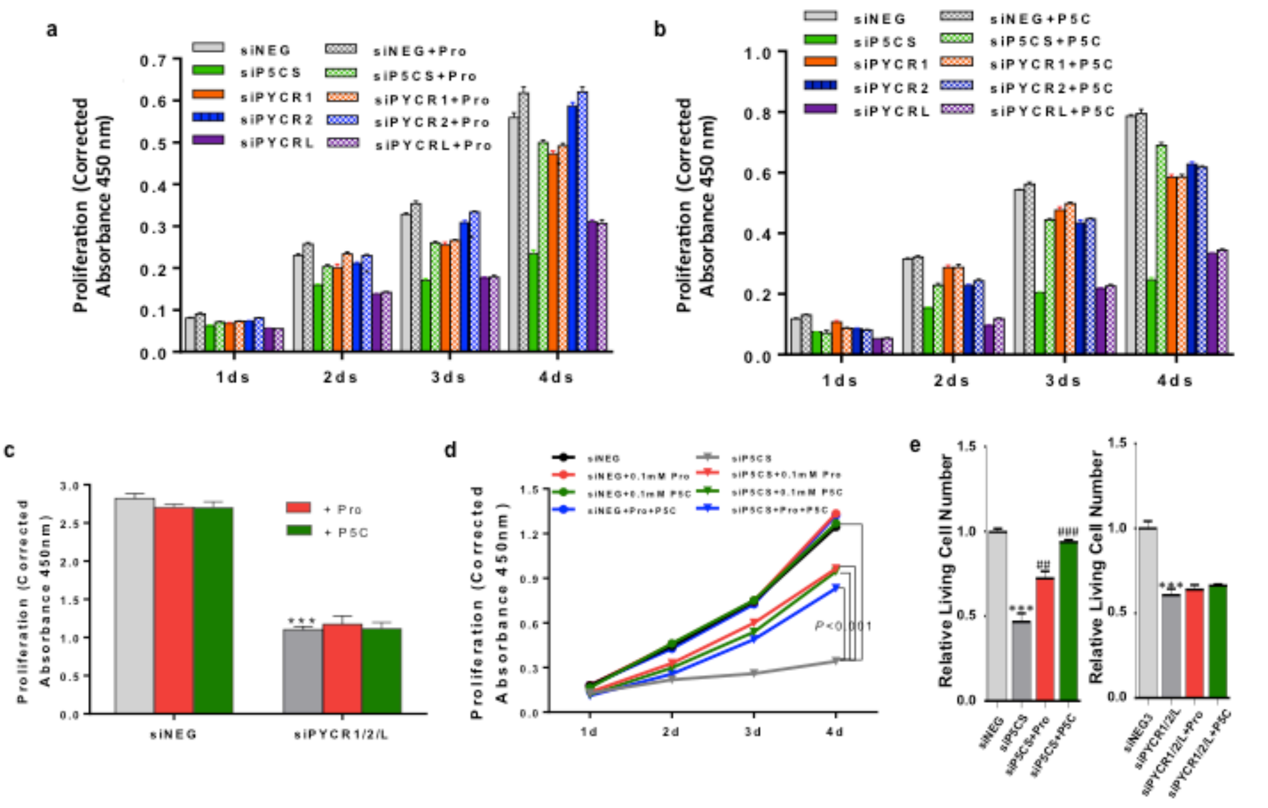

Supplementary Figure 4: Addition of either proline or P5C mitigated the decreased cell growth due to P5CS knockdown. (**a-b**) PC9 cells were cultured for 1 - 4 ds after transfected with siNEG, siP5CS or siPYCR 1, 2 or L, with or without the addition of 0.1 mM proline (**a**) or 0.1 mM P5C (**b**). (**c**) PC9 cells were transfected with siPYCR 1, 2 and L simultaneously. 0.5 mM proline or P5C was added. The proliferation data at 4 days was shown. (**d**) PC9 cell proliferation was shown after treated with indicted conditions. (**e**) P493 lymphoma MYC tet-off cells were cultured under the indicated conditions for 4ds, and the relative living cell number was determined by trypan blue exclusion assay in P493 cells. Data shown are mean ±S.E.M. (n=3). *P* values were obtained by two-way ANOVA in **a**, **b**, and **d**, and two-tailed Student's *t*-test in **c** and **e**. $^{***}P<0.001$ compared with siNEG control group， $^{\#\#}P<0.01$,$^{\#\#\#}P<0.001$, compared with siP5CS group.
